# TAZ and YAP are frequently activated oncoproteins in sarcomas

**DOI:** 10.18632/oncotarget.8979

**Published:** 2016-04-25

**Authors:** Colleen A. Fullenkamp, Sarah L. Hall, Omar I. Jaber, Brittany L. Pakalniskis, Erica C. Savage, Johanna M. Savage, Georgina K. Ofori-Amanfo, Allyn M. Lambertz, Stephanie D. Ivins, Christopher S. Stipp, Benjamin J. Miller, Mohammed M. Milhem, Munir R. Tanas

**Affiliations:** ^1^ Department of Pathology, University of Iowa, Iowa City, IA, USA; ^2^ Department of Biology, University of Iowa, Iowa City, IA, USA; ^3^ Department of Orthopedics and Rehabilitation, University of Iowa, Iowa City, IA, USA; ^4^ Department of Internal Medicine, University of Iowa, Iowa City, IA, USA

**Keywords:** TAZ, WWTR1, YAP, Hippo pathway, sarcoma, Pathology Section

## Abstract

TAZ (*WWTR1*) and YAP are transcriptional coactivators and oncoproteins inhibited by the Hippo pathway. Herein we evaluate 159 sarcomas representing the most prevalent sarcoma types by immunohistochemistry for expression and activation (nuclear localization) of TAZ and YAP. We show that 50% of sarcomas demonstrate activation of YAP while 66% of sarcomas demonstrate activated TAZ. Differential activation of TAZ and YAP are identified in various sarcoma types. At an RNA level, expression of *WWTR1* or *YAP1* predicts overall survival in undifferentiated pleomorphic sarcoma and dedifferentiated liposarcoma. Immunohistochemistry demonstrates that TAZ and YAP expression and activation are positively correlated with grade in the well-differentiated liposarcoma to dedifferentiated liposarcoma tumor progression sequence as well as conventional chondrosarcomas. TAZ and YAP are constitutively activated oncoproteins in sarcoma cell lines. Knock-down of TAZ and YAP demonstrate differential activity for the two proteins. Verteporfin decreases colony formation in soft agar as well as *CTGF* expression in sarcoma cell lines harboring activated TAZ and YAP.

## INTRODUCTION

TAZ(*WWTR1* is the gene) and YAP are transcriptional coactivators and paralogues of one another normally inhibited by the Hippo pathway, a developmentally important signal transduction pathway that limits tissue growth and organ size. [1, 2] The Hippo pathway is a series of core serine/threonine kinases including the STE20-like protein kinases 1 and 2 (MST1/2) [3–6] and the large tumor suppressor 1 and 2 (LATS1/2) [7, 8]. They form a complex with the MOB1A/B [9] and Salvador proteins [4] [6] which form a scaffold for the above kinases. When the MST and LATS kinases are activated by upstream signals, the LATS kinases phosphorylate TAZ/YAP on several serines causing translocation of TAZ and YAP from the nucleus into the cytoplasm where they subsequently undergo ubiquitin-mediated degradation and inactivation. [1, 2]

Recently, TAZ and YAP have emerged as important oncoproteins in a number of different carcinomas including breast, colon, liver, lung, pancreas, and thyroid cancers [10–17]. As transcriptional coactivators, TAZ and YAP are constitutively activated and located within the nucleus in the above cancers. Unexpectedly, mutations in the Hippo pathway or TAZ/YAP are rare [10]. One exception is epithelioid hemangioendothelioma (EHE), an endothelial cell sarcoma, which has been shown to harbor *WWTR1-CAMTA1* (90% of tumors) [18, 19] and *YAP1-TFE3* (10% of tumors) [20] gene fusions. The two gene fusions are the only consistent genetic alterations of *WWTR1* and *YAP1* in a cancer. Fusion of CAMTA1 to TAZ in the TAZ-CAMTA1 fusion protein constitutively activates the N terminus of TAZ by negating inhibitory effects of the Hippo pathway. [21]

The observation that the *WWTR1-CAMTA1* and *YAP1-TFE3* gene fusions occur in a sarcoma suggests these cancers are particularly susceptible to perturbations within the Hippo pathway. This is supported by other studies that demonstrate TAZ and YAP stimulate the development of certain connective tissues/organs and are oncogenic drivers of mesenchymal neoplasms. A Lats1 knock-out mouse develops sarcomas, but not carcinomas or lymphomas [22]. TAZ has been shown to represent an important molecular rheostat in mesenchymal stems cells, regulating differentiation along adipocytic and osteogenic lineages [23]. Other lines of evidence accumulating over the past few years have further implicated YAP as an oncoprotein involved in sarcomagenesis. A mouse model of embryonal rhabdomyosarcoma was established by expressing human YAP S127A in satellite skeletal muscle cells. [24] YAP has also been shown to function as an oncoprotein downstream of the *PAX3-FOXO1* gene fusion in alveolar rhabdomyosarcoma [25]. Recently it was shown that YAP complexes with FOXM1 to drive tumorigenesis in undifferentiated pleomorphic sarcoma and liposarcoma [26].

The above studies have shown that YAP is an oncoprotein mainly in the tumorigenesis of rhabdomyosarcoma, and to some degree, undifferentiated pleomorphic sarcoma and liposarcoma. However sarcomas are comprised of a heterogeneous group of sarcomas, over 50 different histological types have been described [27], and a comprehensive evaluation of YAP's expression and activation in these other sarcoma types has not been investigated. Furthermore, with the exception of the TAZ-CAMTA1 fusion protein in EHE, TAZ's expression and activation in sarcomas has been unexplored. Herein, we comprehensively evaluate the expression and activation of TAZ and YAP in over 150 sarcomas, explore the relative contribution of TAZ and YAP to sarcomagenesis, as well as the viability of inhibiting the TAZ/YAP-TEAD4 complex in sarcomas.

## RESULTS

### TAZ/YAP expression in sarcomas is correlated with decreased overall survival

To confirm the hypothesis that TAZ and YAP are oncoproteins in the pathogenesis of sarcomas, we evaluated data from The Cancer Genome Atlas (TCGA). The sarcoma (SARC) gene expression (IlluminaHiSeq) data set was comprised of 264 sarcomas at the time of evaluation. Grouping of all 264 sarcomas together including various histological types of sarcoma did not demonstrate a correlation between *WWTR1* and *YAP1* expression and overall survival (data not shown). Different histological types of sarcomas demonstrate different clinical behavior, suggesting that grouping different types of sarcomas together was obscuring the results. To evaluate this possibility, we performed survival analysis individually on two of the most common types of sarcoma included in the TCGA data set, undifferentiated pleomorphic sarcoma (UPS) and dedifferentiated liposarcomas (DDLPS).

**Figure 1:**
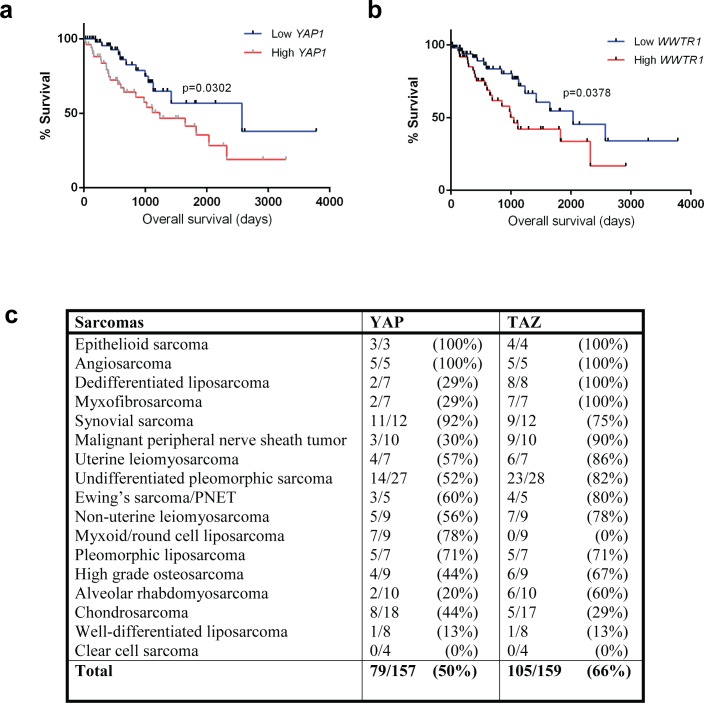
Survival data derived from The Cancer Genome Atlas for undifferentiated pleomorphic sarcoma and dedifferentiated liposarcoma (combined) **a.** High *YAP1* expression results in reduced survival (*p* = 0.03). **b.** High *WWTR1* expression results in reduced survival (*p* = 0.04). **c.** Table summarizing TMA data demonstrating expression/activation in different histological sarcoma types.

A total of 55 dedifferentiated liposarcomas and 50 undifferentiated pleomorphic sarcomas were initially evaluated separately. DDLPS and UPS were divided into two equivalent groups that expressed either high or low *WWTR1* and *YAP1.* (High levels of *WWTR1*/*YAP1* were defined as the upper 50% percentile, while low levels were defined as the lower 50% percentile). Kaplan-Meier analysis performed on these two sarcoma types individually demonstrated a correlation between increased *WWTR1* and *YAP1* expression and reduced survival, however this correlation did not reach statistical significance because the number of cases present were insufficient to power the analysis (data not shown). To address this, we combined the UPS and DDLPS data sets since these sarcomas exhibit similar clinical behavior. This analysis showed that increased *WWTR1* (*p* = 0.0378) or *YAP1* (*p* = 0.0302) expression correlated with reduced survival (Figure 1a and 1b). Sarcomas expressing high levels of *WWTR1* had a median survival of 2.9 years, whereas those with low levels of *WWTR1* expression had a median survival of 5.6 years. Similarly, sarcomas expressing high levels of *YAP1* had a median survival of 3.4 years, whereas those with low levels of YAP1 had a median survival of 7.1 years. These findings indicate TAZ and YAP are oncoproteins which drive disease progression in sarcoma patients.

### TAZ and YAP are commonly activated in sarcomas

Full-length YAP had been shown to function as an oncoprotein in rhabdomyosarcomas, undifferentiated pleomorphic sarcomas, and liposarcomas [24] [25] [26]. It was unknown whether YAP is more broadly activated in other sarcoma types. In addition, the function of full-length TAZ had not been previously investigated in sarcomas. To investigate whether TAZ is activated in sarcomas and determine whether YAP is more broadly activated in other sarcomas, we constructed a tissue microarray (TMA) of 159 primary sarcomas. Essentially all of the most common sarcoma types were arrayed (Figure 1c).

Immunohistochemistry for TAZ and YAP was performed on the above TMA (Figure 1c). Evaluating protein expression in this way allowed for simultaneous evaluation of expression levels as well as localization. Since they are transcriptional co-activators, nuclear localization of TAZ and YAP serves as an indicator of their activation [2, 28]. Tumors were scored according to the percentage of cells that harbored activated (nuclear) TAZ and YAP as well as the intensity of staining (see Methods and Materials for details).

**Figure 2:**
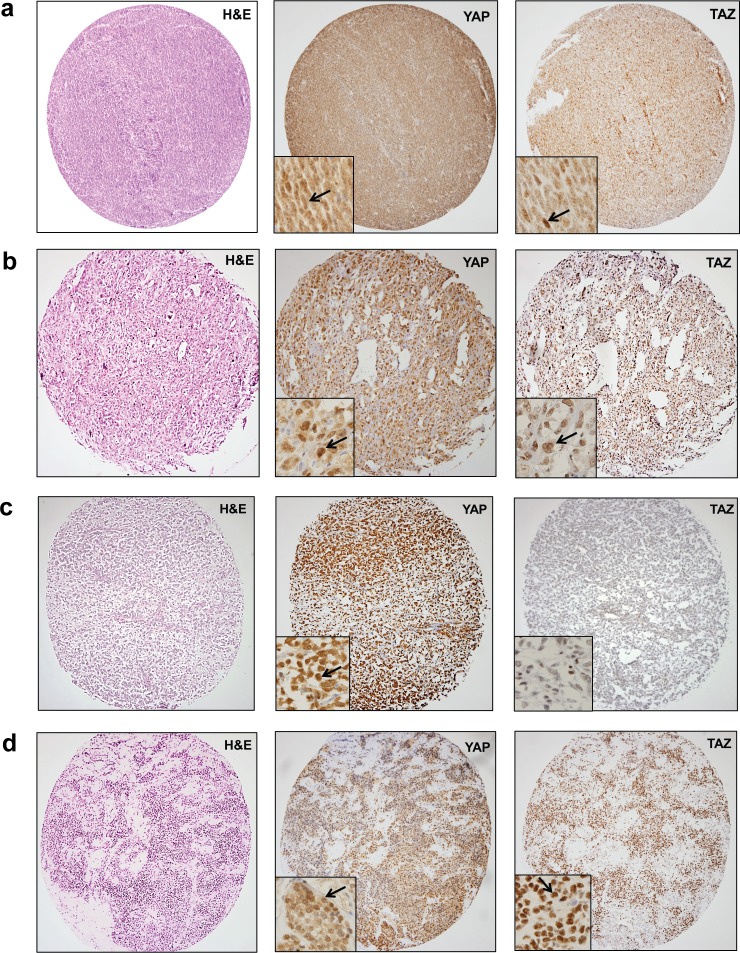
YAP and TAZ are widely expressed and activated (nuclear localization) across multiple histological sarcoma types Higher power magnification (400X) demonstrates nuclear localization (see inset boxes and arrows). **a.** Synovial sarcoma with expression and nuclear localization of both YAP and TAZ. Synovial sarcomas typically exhibited nuclear localization of both YAP and TAZ. **b.** Undifferentiated pleomorphic sarcoma with activated YAP and TAZ. **c.** Myxoid/round cell liposarcoma showing diffuse/strong expression and nuclear localization of YAP. Essentially no TAZ expression/activation is present. **d.** Alveolar rhabdomyosarcoma with expression and nuclear localization of YAP as well as TAZ. Rhabdomyosarcomas were more likely to exhibit activated TAZ (60%) than activated YAP (20%).

Overall, 2/3 of sarcomas harbored activated TAZ (66%), while half of them demonstrated activated YAP (50%) (Figure 1c). In some sarcoma types, all of the sarcomas assayed demonstrated activated TAZ or YAP including epithelioid sarcoma, angiosarcoma, dedifferentiated liposarcoma, and myxofibrosarcoma. TAZ or YAP were activated in the majority of some sarcomas including synovial sarcoma (92%) (Figure 2a), malignant peripheral nerve sheath tumor (90%), uterine leiomyosarcoma (86%), undifferentiated pleomorphic sarcoma (82%) (Figure 2b), Ewing's sarcoma/PNET (80%), non-uterine leiomyosarcoma (78%), myxoid/round cell liposarcoma (78%) (Figure 2c), pleomorphic liposarcoma (71%), high grade osteosarcoma (67%), and alveolar rhabdomyosarcoma (60%) (Figure 2d). Less than half of chondrosarcomas (44%) and well-differentiated liposarcomas (13%) harbored activated TAZ/YAP. The relatively low percentage of chondrosarcomas and well-differentiated liposarcomas harboring activated TAZ and YAP is due to the observation (discussed further below) that the activated oncoproteins are relatively absent in the lower grade examples of these sarcomas (grade 1/2 chondrosarcomas and well-differentiated liposarcoma), but present at higher frequencies in higher grade examples (grade 3 chondrosarcoma and dedifferentiated liposarcoma). In the four cases evaluated, clear cell sarcoma of soft parts did not demonstrate activated TAZ and YAP, indicating that activation of TAZ and YAP was not a non-specific finding (Figure 1c).

### TAZ and YAP are differentially activated in certain sarcoma types

Previous study of the Hippo-TAZ/YAP axis in sarcomas has focused predominantly on wild-type YAP and with the exception of study of the TAZ-CAMTA1 fusion protein has largely ignored the effect of full-length TAZ. Although the numbers included were small, all of the epithelioid sarcomas and angiosarcomas in the TMA were demonstrated to be positive for activated TAZ and YAP. A high percentage of synovial sarcomas exhibited both activated YAP (92%) and TAZ (75%) (Figure 1c). In contrast, several other sarcomas demonstrated predominantly activated TAZ including myxofibrosarcoma (100%), dedifferentiated liposarcoma (100%), malignant peripheral nerve sheath tumor (90%), uterine/non-uterine leiomyosarcoma (86 and 78% respectively), and undifferentiated pleomorphic sarcoma (82%). Myxoid/round cell liposarcoma was unique in that it preferentially demonstrated activation of YAP (78%) and not TAZ (0%). This strongly suggested that despite the fact that TAZ and YAP share a high degree of homology, and are assumed to function in the same way, this may not necessarily be the case. Overall, TAZ (66%) was more frequently activated than YAP (50%), suggesting that in this survey of various sarcomas, TAZ rather than YAP was the more commonly activated oncoprotein (*p* = 0.0061) (Figure 1c).

### TAZ and YAP activation is associated with increased grade/tumor progression in sarcomas

TAZ and YAP have been implicated in tumor progression in other cancers (e.g. breast cancer) [29]. To test the hypothesis that TAZ/YAP are involved in tumor progression in sarcomas, we evaluated TAZ and YAP activation in two sarcomas with a well-established tumor progression sequence.

**Figure 3:**
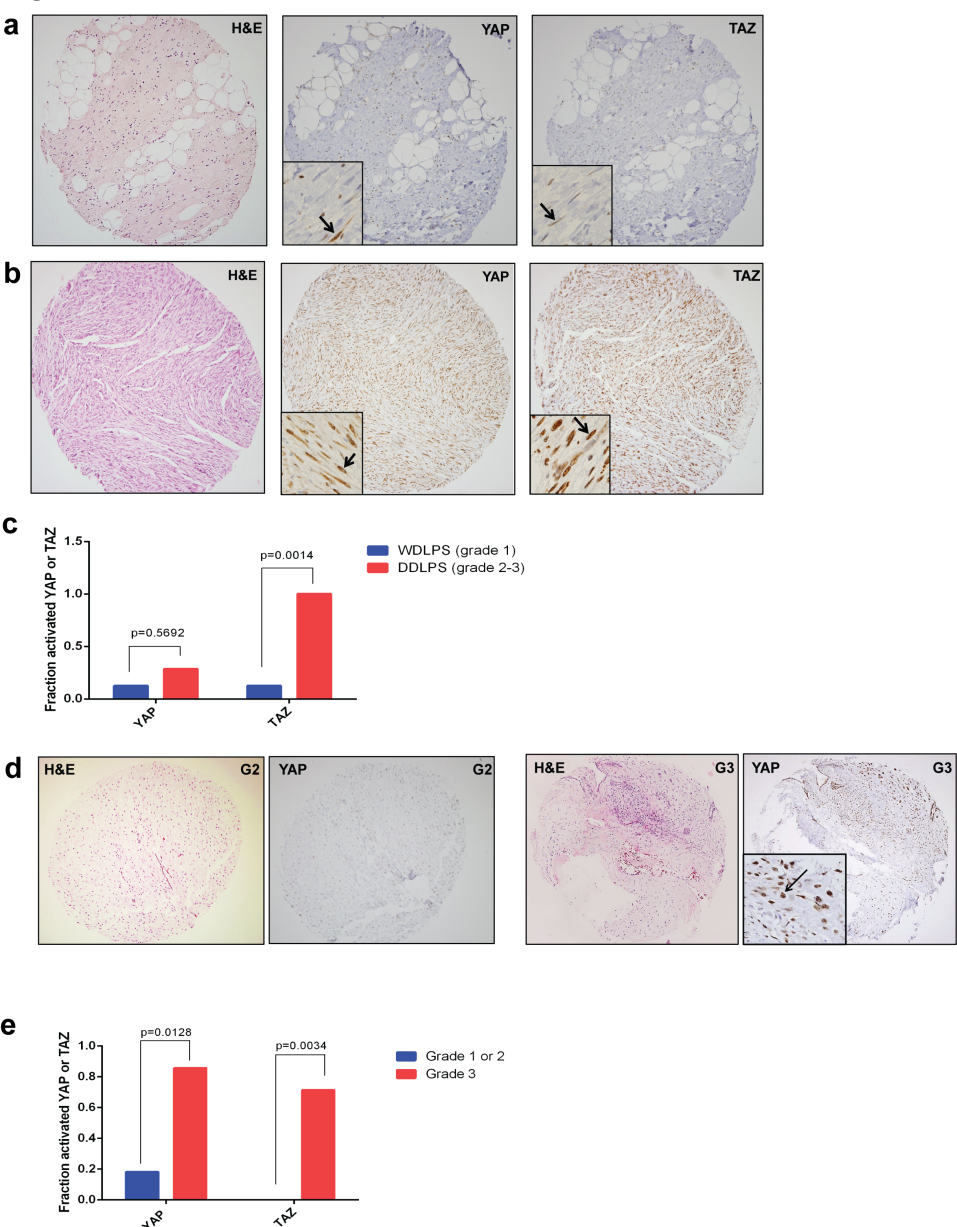
YAP and TAZ are associated with increased grade **a.** TAZ/YAP are only focally and weakly expressed in well-differentiated liposarcoma (grade 1 tumors). **b.** Increased YAP and TAZ expression in dedifferentiated liposarcoma (grade 2 to 3). **c.** Graphical representation of YAP and TAZ activation in dedifferentiated liposarcoma (DDLPS) *vs*. well-differentiated liposarcoma (WDLPS); TAZ is activated to a greater degree in dedifferentiated liposarcoma as compared to well-differentiated liposarcoma (*p* = 0.0014). **d.** YAP and TAZ activation levels are low to absent in grade 1 and 2 chondrosarcomas, but present in most (~70-80%) of grade 3 chondrosarcomas **e.** Graphical representation of YAP and TAZ activation in chondrosarcoma, showing a statistically significant increase of activated YAP and TAZ is grade 3 chondrosarcoma as compared to grade 1/2 chondrosarcomas (YAP, *p* = 0.0128) and (TAZ, *p* = 0.0034).

The well-differentiated liposarcoma-dedifferentiated liposarcoma sequence is amenable to grading by the French Federation of Cancer Centers Sarcoma Group (FNCLCC) three tiered grading scheme [30]. Well-differentiated liposarcoma (WDLPS) is a low grade (FNCLCC grade 1 of 3) is a sarcoma exhibiting adipocytic differentiation which causes significant morbidity due to its propensity for local recurrence [31]. After multiple recurrences, or sometimes during the initial presentation, WDLPS can undergo tumor progression to dedifferentiated liposarcoma (DDLPS), a higher grade (grade 2 or 3) sarcoma with a more aggressive clinical course including the ability to metastasize [32]. WDLPS typically contained only a few scattered cells with nuclear localization of TAZ or YAP (Figure 3a). Only 1 of 8 (13%) of well-differentiated liposarcomas was scored as being positive for TAZ or YAP. In contrast, 8 of 8 (100%) of dedifferentiated liposarcomas harbored activated TAZ, a statistically significant increase (*p* = 0.0014) (Figure 3b, 3c). The degree of YAP activation did not change significantly between WDLPS and DDLPS.

We tested this hypothesis in chondrosarcoma, another sarcoma containing a well-defined tumor progression sequence and grading scheme. Conventional chondrosarcomas are stratified according to a 3-tier grading scheme according to their cellularity, degree of differentiation (hyaline cartilage *vs*. myxoid stroma), cytological atypia/morphology, and mitotic activity. Akin to the WDLPS to DDLPS sequence, higher grade chondrosarcomas have a more aggressive clinical course including metastasis [33]. Eighteen chondrosarcomas were included in the tissue microarray. Overall, 8 of 18 chondrosarcomas (44%) harbored activated YAP, while 5 of 17 chondrosarcomas (29%) harbored activated TAZ. When evaluated according to grade, 2 of 11 (18%) grade 1 and 2 chondrosarcomas harbored activated YAP. In contrast, 6 of 7 (86%) grade 3 chondrosarcomas harbored activated YAP (*p* = 0.0128). Similarly, no grade 1 or grade 2 chrondrosarcomas demonstrated activated TAZ, while 5 of 7 grade 3 chondrosarcomas (71%) harbored activated TAZ (*p* = 0.0034) (Figure 3d, 3e). This is consistent with the liposarcoma data, and indicates that at least in some types of sarcoma, TAZ and YAP are oncoproteins which drive tumor progression since their activation is more frequent in higher grades of these sarcomas.

### TAZ and YAP are expressed and constitutively activated in the HT-1080 and SK-LMS-1 sarcoma cell lines

Immunohistochemistry performed on the TMA suggested both TAZ and YAP were oncoproteins activated in a variety of sarcomas. To examine the oncogenic activity of TAZ and YAP *in vitro,* we performed several studies utilizing the SK-LMS-1 (leiomyosarcoma) and HT-1080 (fibrosarcoma) cell lines.

Previous studies of the TAZ-CAMTA1 fusion protein demonstrated that it functioned as an oncoprotein because the TAZ portion of the fusion protein was constitutively activated and no longer inhibited by the Hippo pathway [21]. The Hippo pathway is activated during cell confluence and normally causes a shift in the localization of TAZ and YAP from the nucleus into the cytoplasm. TAZ-CAMTA1 was demonstrated to be free from regulation by the Hippo pathway *via* its constitutive nuclear localization, even during cell confluence [21]. Similar findings have been demonstrated utilizing the TAZ 4SA and YAP 5SA constructs which substitute all serines phosphorylated by the Hippo pathway [34, 35]. By extension, if full-length TAZ and YAP are truly oncoproteins in sarcomas, they should remain in the nucleus even during conditions of cellular crowding.

**Figure 4:**
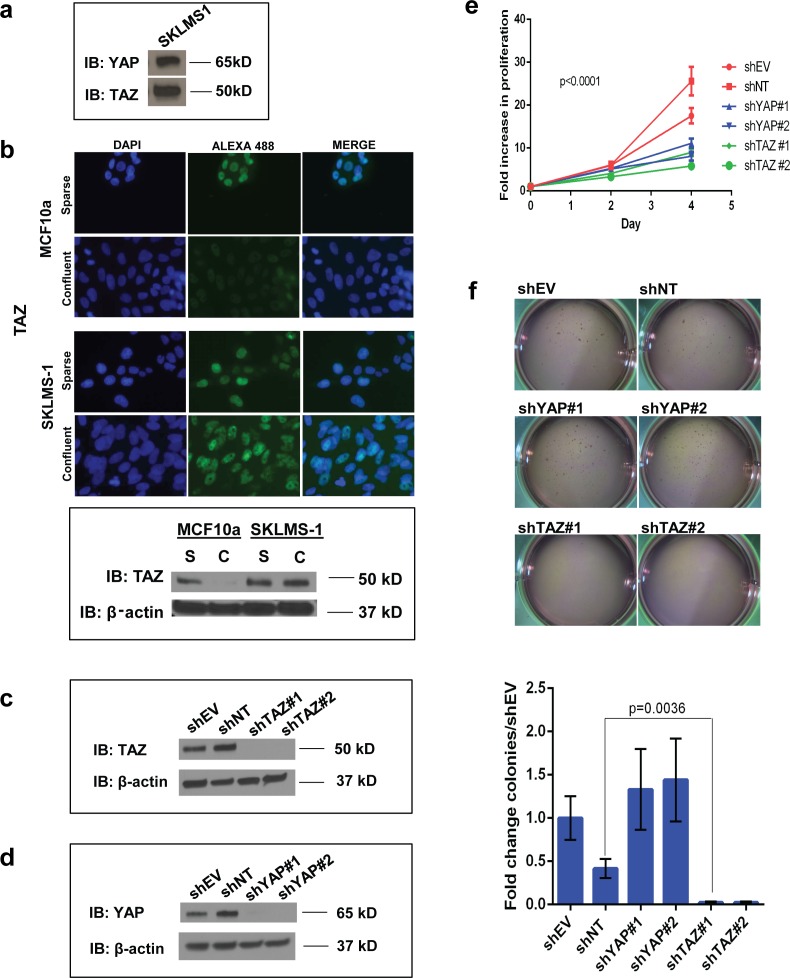
*In vitro* studies demonstrating that TAZ and YAP are activated oncoproteins in the SK-LMS-1 cell line **a.** Western blot demonstrating YAP and TAZ expression in SK-LMS-1. **b.** Immunofluorescence shows TAZ is localized within the nucleus of SK-LMS-1 cells and MCF10a cells (negative control) when plated at sparse conditions and the Hippo pathway is inactivated. During confluent conditions, the Hippo pathway is activated resulting in an almost complete absence of signal for TAZ in MCF10a cells. In contrast, in SK-LMS-1 cells, TAZ is constitutively activated and remains localized within the nucleus during confluent conditions. The absence of signal of TAZ in MCF10a cells during cell confluence is due to degradation and confirmed by western blot; note that by comparison TAZ levels in SK-LMS-1 cells are relatively constant. *S* = sparse, *C* = confluent for Western blot. **c.** TAZ is effectively knocked-down in SK-LMS-1 cells with two shRNA constructs. **d.** YAP is effectively knocked-down in SK-LMS-1 cells with two shRNA constructs. **e.** Knock-down of TAZ and YAP in SK-LMS-1 cells reduces proliferation (*p* < 0.0001). **f.** Knock-down of TAZ, but not YAP, reduces colony formation in soft agar (*p* = 0.0036).

To test this hypothesis, we evaluated cellular localization of TAZ and YAP in two sarcoma cell lines, the SK-LMS-1 (leiomyosarcoma) and HT-1080 (fibrosarcoma) cell lines. Both TAZ and YAP were expressed by both the SK-LMS-1 cell line (Figure 4a) and HT-1080 cell lines (Figure S2, S3a). Localization was evaluated by immunofluorescence utilizing MCF10a cells as a control, since the normal regulation of TAZ and YAP by the Hippo pathway has been well documented in this immortalized but non-transformed cell line [2, 34]. Under sparse conditions, TAZ is localized within the nucleus of both SK-LMS-1 cells as well as MCF10a cells, as would be expected since the Hippo pathway is inactivated (Figure 4b). When grown to confluence, TAZ expression in MCF10a cells was essentially absent, confirmed by Western blot (Figure 4b). This is consistent with the Hippo pathway activation, translocation of TAZ from the nucleus into the cytoplasm, and subsequent degradation as has been previously described [36]. In contrast, SK-LMS-1 cells grown to confluence showed no significant change in TAZ nuclear localization by immunofluorescence, and overall expression was unchanged as confirmed by Western blot (Figure 4b).

### TAZ and YAP promote colony formation and proliferation in soft agar

To confirm that TAZ and YAP were oncoproteins in the above cell lines, we evaluated the effect of shRNA knock-down of TAZ and YAP by assaying various hallmarks of cancer including proliferation and anchorage-independent growth. Knock-down of TAZ and YAP were achieved in both the SK-LMS-1 (Figure 4c, 4d) and HT-1080 cell lines (Figure S3a). Knock-down of TAZ markedly decreased proliferation of the SK-LMS-1 cell line. Loss of YAP expression also reduced proliferation to a somewhat lesser degree (Figure 4e). However, while knock-down of TAZ resulted in complete abrogation of colony formation in soft agar, loss of expression of YAP did not have an effect of colony formation in soft agar (Figure 4f).

A similar trend was identified with the HT-1080 cell lines. Knock-down of TAZ caused a mild, but statistically significant decrease in proliferation, while knock-down of YAP did not significantly decrease proliferation (Figure S3b, S3c). However, knock-down of TAZ and YAP both significantly decreased colony formation in soft agar (Figure S3d).

The discordance between TAZ/YAP knock-down on proliferation and anchorage independent growth in both the SK-LMS-1 and HT1080 cell lines suggest that TAZ and YAP activate distinct transcriptional programs mediating anchorage-independent growth and proliferation that are cell line and sarcoma type-dependent. YAP loss caused a decrease in proliferation with the SK-LMS-1 cell line, while it caused a decrease in anchorage independent growth with the HT1080 cell line. TAZ, on the other hand, was responsible for driving proliferation and anchorage independent growth in both lines. While most studies evaluating the Hippo pathway in sarcomas have focused on YAP [24, 25] [26], the above data suggest TAZ is the dominant oncoprotein in at least some sarcomas. This is corroborated by the above TMA data set which identified a number of sarcomas in which TAZ rather than YAP was activated, and which showed that overall, TAZ was more commonly activated than YAP (Figure 1c).

### TAZ and YAP can be therapeutically targeted in sarcoma cell lines

Since TAZ and YAP do not contain DNA binding domains of their own, they require complexing with other transcription factors which do contain DNA binding domains [28, 37]. Members of the TEAD transcription factor family have been implicated as the predominant transcription factors which mediate the oncogenic transcriptional programs of TAZ and YAP [38] [39] [40]. As has been shown previously, the YAP-TEAD4 complex can be inhibited pharmacologically with verteporfin (Visudyne ^®^), a heme analog [41]. It has been demonstrated it can disrupt the YAP-TEAD4 complex in sarcomas where YAP is the oncogenic driver and thus inhibit proliferation [26]. It was unknown whether verteporfin could inhibit colony formation in soft agar, and whether it would be effective in a cell line such as SK-LMS-1 in which TAZ is the dominant oncoprotein.

**Figure 5:**
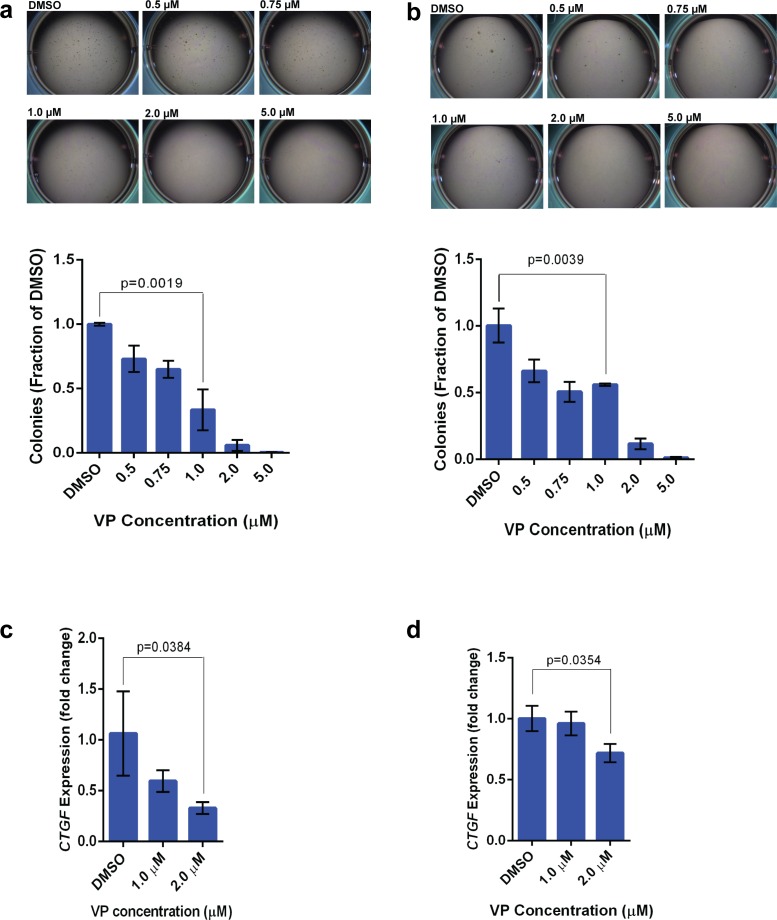
Verteporfin inhibits TAZ/YAP activity in sarcoma cells lines and abrogates colony formation in soft agar Verteporfin reduces colony formation in a dose dependent manner in the HT-1080 **a.** and SK-LMS-1 cell lines **b.** Administration of verteporfin results in decreased expression of *CTGF* (a canonical transcriptional target of the TEAD transcription factors) in a dose dependent manner in both the HT-1080 **c.** and SK-LMS-1 cell lines **d.**

To test the potential efficacy of verteporfin, we plated HT-1080 cells in soft agar with concentrations of verteporfin (VP) which ranged from 0.5μM to 5μM. A marked decrease in colony formation as compared to the DMSO control was identified at 0.75μM, with almost complete abrogation of colony formation at 1-2 μM (Figure 5a). Similarly, verteporfin resulted in decreased colony formation in SK-LMS-1 cells at 0.75 μM (Figure 5b), with almost complete abrogation of colony formation at 2 μM. Essentially no decrease in proliferation over three days was identified at concentrations of VP up to 1 μM (data not shown), arguing against the possibility its effect in soft agar was due to non-specific toxicity of the drug.

Connective tissue growth factor (*CTGF*) is a canonical read-out of the TAZ/YAP transcriptional program. TAZ and YAP complex with TEAD4 which in turn binds to the *CTGF* promoter [40]. To confirm VP is functioning by disrupting the TAZ/YAP-TEAD4 complex, we evaluated *CTGF* expression in the SK-LMS- 1 and HT1080 sarcoma cell lines as a function of different concentrations of VP. Quantitative RT-PCR showed a dose-dependent reduction in *CTGF* levels in both the HT-1080 cell line (Figure 5c) as well as the SK-LMS-1 cell line (Figure 5d), concomitant to the decrease in colony formation in soft agar. Verteporfin reduces *CTGF* expression in HT-1080 cells to a greater degree than in SK-LMS-1 cells. This is consistent with the greater role YAP plays in HT-1080 cells as compared to SK-LMS-1 cells; verteporfin was initially identified as a molecule interrupting the YAP-TEAD4 complex and is likely less effective at interrupting TAZ-TEAD4 binding since the critical interactions of YAP and TAZ with TEAD4 occur *via* different residues [42].

## DISCUSSION

Herein, we performed a comprehensive evaluation of major sarcoma types and showed that approximately 2/3 of sarcomas demonstrate activated TAZ, while about half of sarcomas demonstrate activated YAP. Activation of TAZ and YAP were identified across several histological types, regardless of line of differentiation.

Utilizing TCGA data, RNA levels of *WWTR1* and *YAP1* were demonstrated to be inversely correlated with overall survival in dedifferentiated liposarcoma and undifferentiated pleomorphic sarcomas. At the protein level, expression and activation of TAZ and YAP were shown to correlate with increased grade in the well-differentiated to dedifferentiated liposarcoma tumor progression sequence and conventional chondrosarcoma. The above findings indicate the potential for activated TAZ and YAP to be utilized as prognostic biomarkers in sarcomas. Additional studies are warranted to validate utilization of TAZ and YAP as prognostic biomarkers in larger data sets. While evaluation of TAZ and YAP activation may not replace current grading schemes of sarcomas, we anticipate it will represent a useful adjunct to further modulate prognosis.

Immunofluorescence studies on sarcoma cell lines also demonstrated constitutive activation of TAZ and YAP. TAZ and YAP were constitutively localized within the nucleus even during cell confluence when the Hippo pathway is activated. shRNA knock-down of both TAZ and YAP resulted in abrogation of various hallmarks of cancer.

Most studies of the Hippo pathway in sarcomas have focused on activated YAP [24–26]. Although the overall assumption is that TAZ and YAP function in the same way, we show that TAZ and YAP are differentially activated in sarcomas and demonstrate different functions *in vitro*. Some sarcomas (e.g. myxoid/round cell liposarcoma), appear to be entirely dependent upon YAP activation for their pathogenesis. However, in other sarcomas, such as myxofibrosarcoma or malignant peripheral nerve sheath tumor, TAZ is the more commonly activated oncoprotein. Overall, in the sarcomas evaluated in the TMA, TAZ was more commonly activated than YAP in these sarcomas, reaching statistical significance. Knock-down experiments performed in the SK-LMS-1 and HT-1080 cell lines showed that TAZ drove more hallmarks of cancer than YAP. Additional studies are warranted to evaluate the differential activities of TAZ and YAP in different types of sarcomas as well as other cancers.

**Figure 6:**
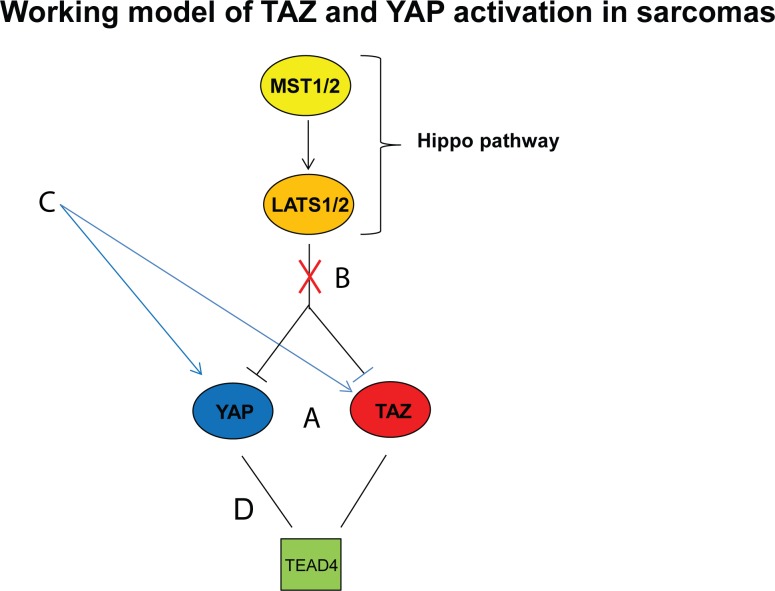
Working model of YAP and TAZ activation in sarcomas **a.** YAP and TAZ have differential effects in different sarcomas as demonstrated in clinical samples and cell lines. YAP and TAZ are constitutively activated (localized within the nucleus) due to silencing of the Hippo pathway **b.** or activation of still unknown **c.** upstream pathways. **d.** The complex between YAP/TAZ and TEAD4 can be inhibited pharmacologically, indicating YAP/TAZ represent therapeutic targets in sarcomas.

In summary, we showed that TAZ and YAP are commonly activated oncoproteins in sarcomas. They are differentially activated in various sarcomas and do not functionally phenocopy each other in the two sarcoma cell lines evaluated. They are constitutively activated either due to inhibition of the Hippo pathway as has been suggested by some [25] [26] or by other mechanisms which have been recently delineated including microRNAs [43] and G-proteins/G protein coupled receptors [44] [45] [46]. Finally, we have shown that TAZ and YAP can be pharmacologically inhibited by verteporfin in sarcomas, proof of principle that TAZ and YAP represent therapeutic targets in a number of sarcomas (Figure 6). Recently drugs such as dasatinib and statins, have been shown to effectively target upstream pathways and indirectly inhibit TAZ and YAP activity [47], indicating inhibition of TAZ and YAP may soon become clinically feasible. Although additional studies are required, we anticipate that the Hippo-TAZ/YAP axis will become actionable therapeutically and utilized prognostically across various histological types of sarcoma.

## MATERIALS AND METHODS

### Bioinformatics analysis

Data from The Cancer Genome Atlas (TCGA) was evaluated *via* the University of California Santa Cruz Cancer Genomics browser. The final evaluation was performed on the data set available on 1/6/2016. The sarcoma (SARC) gene expression (IlluminaHiSeq) data set was comprised of 264 sarcomas at the time of evaluation. Undifferentiated pleomorphic sarcoma was defined as sarcomas which had been entered into the TCGA data base with the diagnosis of undifferentiated pleomorphic sarcoma, pleomorphic malignant fibrous histiocytoma, and giant cell malignant fibrous histiocytoma (these have all been grouped together under the classification of undifferentiated pleomorphic sarcoma according to the most recent WHO classification) [27]. High levels of *WWTR1*/*YAP1* were defined as the upper 50% percentile, while low levels were defined as the lower 50% percentile.

### Tissue microarray construction

A total of 159 primary, untreated sarcomas were obtained from the University of Iowa Department of Pathology with previous approval from the Institutional Review Board. The tissue microarray was constructed by arraying the sarcomas in duplicate consisting of 1.0-mm cores taken from formalin fixed paraffin embedded tissue and assembled using a MTA-1 tissue arrayer from Beecher Instruments (Sun Prarie, WI). The mesenchymal neoplasms utilized in the array were reviewed by multiple pathologists (M.R.T., O.I.J, B.L.P., E.C.S., and J.M.S.) and classified according to World Health Organization criteria [27].

### Antibodies

Anti-TAZ (mouse monoclonal 1H9; catalog # LS-C173295) utilized for immunohistochemistry (1:50) and immunofluorescence (1:100) was obtained from LifeSpan BioSciences (Seattle, WA, USA). Anti-YAP (rabbit polyclonal, catalog #sc-15407) utilized for immunohistochemistry (1:100) and immunofluorescence (1:100) was obtained from Santa Cruz Biotechnology (Santa Cruz, CA, USA). Anti-TAZ (rabbit polyclonal, catalog# HPA007415) utilized for Western blot was obtained from Sigma-Aldrich (St. Louis, MO, USA). Anti-YAP (D8H1X XP; catalog #14074) utilized for Western blot was obtained from Cell Signaling (Danvers, MA, USA). β-actin (AC-15; catalog #A5441) was obtained from Sigma-Aldrich. Alexa Fluor 488-conjugated secondary antibody (catalog #A11034, and A11029) was obtained from Invitrogen-Life Technologies (Grand Island, NY, USA). Horseradish peroxidase-conjugated secondary antibodies were obtained from Santa Cruz Biotechnology.

### Evaluation of TAZ and YAP activation by immunohistochemistry

Blood vessels in the tumors were used as an internal control (endothelial cells have previously been shown to highly express TAZ/YAP) (Figure S1b) [19]. Intensity of staining was classified as strong (equivalent to staining within blood vessels), intermediate, and weak. Tumors were defined as being positive if greater than 70% of the cells demonstrated intermediate levels of activated (nuclear) TAZ or YAP.

### Western blot

Harvested cells were lysed in radioimmunoprecipitation assay (RIPA) complete lysis buffer with the addition of Complete Protease Inhibitor Cocktail (Roche) and PhosSTOP Phosphatase Inhibitor Cocktail (Roche) according to the manufacturer's instructions. Total protein concentration was measured using BCA^TM^ Protein Assay Kit (Pierce). Proteins were transferred to a polyvinylidene difluoride (PVDF) membrane and probed with anti-TAZ polyclonal antibody (2:000), anti-YAP polyclonal antibody (1:1000), and β-actin (1:5000).

### Immunofluorescence staining

SK-LMS-1 and HT1080 cells were fixed with 4% paraformaldehyde (in 1X PBS) for 15 min. After washing with PBS, cells were permeabilized and blocked with 0.3% Triton X-100 and 3% fetal bovine serum for 30 min. Cells were incubated with anti-TAZ and anti-YAP antibody diluted (1:100) in 3% fetal bovine serum at 4°C overnight in a humidity chamber. The primary antibody was removed, cells washed, then incubated with Alexa Fluor 488-conjugated secondary antibody (Invitrogen-Life Technologies) for 45 minutes to 1 hr at room temperature. Immunofluorescence was visualized using the Leica DM IL LRD fluorescence microscope, DFC3000 G camera, and AF6000 Modular System software (Leica Microsystems, GmbH, Wetzlar, Germany).

### Cell culture, transfection, and lentiviral transduction

SK-LMS1 and HT-1080 cell lines were obtained from the American Type Culture Collection (ATCC, Manassas, VA, USA) and were cultured in DMEM containing 10% Fetal Bovine Serum (Invitrogen-Life Technologies) and 50μg/mL pen/strep. All cells were cultured at 37°C and 5% CO_2_. Verteporfin was dissolved in dimethyl sulfoxide (DMSO) and subsequently diluted in media to the indicated concentrations.

### TAZ/YAP RNA interference-mediated silencing and lentiviral transduction

The following pLKO.1-puromycin constructs were obtained from Sigma-Aldrich. Empty vector construct (SHC001), scrambled negative control (SHC002), and TAZ knock-down constructs TRCN0000319149 (shTAZ#1), TRCN0000319150 (shTAZ#2) (this is shTAZ#1 in Figure 4), TRCN0000319224 (shTAZ#3), TRCN0000370006 (shTAZ#4), TRCN0000370007 (shTAZ#5) (this is shTAZ#2 in Figure 4). YAP knock-down constructs were obtained from Addgene #42540 (shYAP#1), #42541 (shYAP1#2) (this is shYAP#1 in Figure 4), #27368 (pLKO.1 shYAP#3) (this is shYAP#2 in Figure 4). To produce lentivirus, pLKO.1 vectors (see below) were transfected (Lipofectamine with PLUS reagent, Invitrogen-Life Technologies) into HEK 293T cells along with pCMV8.12 and pVSVG packaging plasmids. Supernatant was collected at 48 and 72 hrs after transfection, filtered with 0.45-μm filter and supplemented with 8 μg/mL polybrene. Pooled stable lines were selected for with 1 μg/mL puromycin for two weeks.

### Proliferation assay

500-1000 cells from the SK-LMS-1 and HT1080 cell lines and the derivative knock-down cell lines and controls were plated in a 96 well plate (100 μL, 9 wells for each derivative cell line). Proliferation was measured with the Dojindo Assay Cell Counting Kit 8 (Dojindo Molecular Technologies, Rockville, MD) according to the manufacturer's instructions. Absorbance was measured using the BioTek: Synergy H1 Hybrid Reader (Winooski, VT).

### Soft agar colony formation assay

A 2mL bottom layer of 0.5% low melt agarose (final) diluted in complete growth medium was established. 5×10^3^ cells were suspended in a 2mL of 0.35% low melt agarose (final) diluted in complete growth medium in the top layer.

### Quantitative reverse transcription polymerase chain reaction

Total RNA was isolated from SK-LMS-1 and HT-1080 cells after administration of verteporfin for 12 hours using TRIzol reagent (Invitrogen-Life Technologies). Total RNA was treated with DNase (Invitrogen-Life Technologies), then column purified using the PureLink RNA mini kit (Ambion-Thermo Fisher Scientific). 0.5μg of DNase treated RNA was converted to cDNA using Superscript III Reverse Transcriptase (Invitrogen-Life Technologies) and 250ng of random primers (Promega, Madison, WI USA). PCR amplification was performed in technical triplicates on the Applied Biosystems (ViiA) Real-Time PCR System (Applied Biosystems-Life Technologies). Relative quantitation was performed utilizing the delta-delta C_T_ method and the geometric mean of β-actin and GAPDH C_T_ values as the reference control. The TaqMan Universal PCR Master Mix (Applied Biosystems-Life Technologies) was utilized as well as PrimeTime standard qPCR primer/probe sets from Integrated DNA Technologies (Iowa City, IA, USA). The Taqman based approach utilized the following primers and probes:

GAPDH forward primer: 5′;-ACATCGCTCAGACACCATG-3′;

GAPDH reverse primer: 5′;-TGTAGTTGAGGTCAATGAAGGG-3′;,

GAPDH probe: 5′;-AAGGTCGGAGTCAACGGATTTGGTC-3′;.

β-actin forward primer: 5′;-ACCTTCTACAATGAGCTGCG-3′;,

β-actin reverse primer 5′;-CCTGGATAGCAACGTACATGG-3′;,

β-actin probe: 5′;-ATCTGGGTCATCTTCTCGCGGTTG-3′;.

CTGF forward primer: 5′;-ACCAATGACAACGCCTCC-3′;,

CTGF reverse primer: 5′;-TTGGAGATTTTGGGAGTACGG-3′;

CTGF probe: 5′;-TGCGAAGCTGACCTGGAAGAGAAC-3′;

### Statistics

For soft agar colony formation assays, statistical significance was evaluated with an unpaired two-tailed *t*-test. For proliferation assays, statistical significance was evaluated using fold increase in proliferation at the terminal time point with an unpaired two-tailed t test (95% confidence intervals, p of 0.05). The mean was used to represent the average of technical replicates. Error bars were used to define one s.d. Each experiment was repeated at least twice.

To determine whether two groups were different when two different outcomes were possible (e.g. evaluating TAZ *vs*. YAP activation in sarcomas), the two-tailed Fisher's exact test was utilized.

## SUPPLEMENTARY MATERIAL FIGURES



## References

[1] Dong J, Feldmann G, Huang J, Wu S, Zhang N, Comerford SA, Gayyed MF, Anders RA, Maitra A, Pan D (2007). Elucidation of a universal size-control mechanism in Drosophila and mammals. Cell.

[2] Zhao B, Wei X, Li W, Udan RS, Yang Q, Kim J, Xie J, Ikenoue T, Yu J, Li L, Zheng P, Ye K, Chinnaiyan A, Halder G, Lai ZC, Guan KL (2007). Inactivation of YAP oncoprotein by the Hippo pathway is involved in cell contact inhibition and tissue growth control. Genes & development.

[3] Harvey KF, Pfleger CM, Hariharan IK (2003). The Drosophila Mst ortholog, hippo, restricts growth and cell proliferation and promotes apoptosis. Cell.

[4] Pantalacci S, Tapon N, Leopold P (2003). The Salvador partner Hippo promotes apoptosis and cell-cycle exit in Drosophila. Nature cell biology.

[5] Udan RS, Kango-Singh M, Nolo R, Tao C, Halder G (2003). Hippo promotes proliferation arrest and apoptosis in the Salvador/Warts pathway. Nature cell biology.

[6] Wu S, Huang J, Dong J, Pan D (2003). hippo encodes a Ste-20 family protein kinase that restricts cell proliferation and promotes apoptosis in conjunction with salvador and warts. Cell.

[7] Justice RW, Zilian O, Woods DF, Noll M, Bryant PJ (1995). The Drosophila tumor suppressor gene warts encodes a homolog of human myotonic dystrophy kinase and is required for the control of cell shape and proliferation. Genes & development.

[8] Xu T, Wang W, Zhang S, Stewart RA, Yu W (1995). Identifying tumor suppressors in genetic mosaics: the Drosophila lats gene encodes a putative protein kinase. Development (Cambridge, England).

[9] Lai ZC, Wei X, Shimizu T, Ramos E, Rohrbaugh M, Nikolaidis N, Ho LL, Li Y (2005). Control of cell proliferation and apoptosis by mob as tumor suppressor, mats. Cell.

[10] Harvey KF, Zhang X, Thomas DM (2013). The Hippo pathway and human cancer. Nature reviews Cancer.

[11] Mo JS, Park HW, Guan KL (2014). The Hippo signaling pathway in stem cell biology and cancer. EMBO reports.

[12] Chan SW, Lim CJ, Guo K, Ng CP, Lee I, Hunziker W, Zeng Q, Hong W (2008). A role for TAZ in migration, invasion, and tumorigenesis of breast cancer cells. Cancer research.

[13] Wang L, Shi S, Guo Z, Zhang X, Han S, Yang A, Wen W, Zhu Q (2013). Overexpression of YAP and TAZ is an independent predictor of prognosis in colorectal cancer and related to the proliferation and metastasis of colon cancer cells. PloS one.

[14] Zender L, Spector MS, Xue W, Flemming P, Cordon-Cardo C, Silke J, Fan ST, Luk JM, Wigler M, Hannon GJ, Mu D, Lucito R, Powers S, Lowe SW (2006). Identification and validation of oncogenes in liver cancer using an integrative oncogenomic approach. Cell.

[15] Zhou Z, Hao Y, Liu N, Raptis L, Tsao MS, Yang X (2011). TAZ is a novel oncogene in non-small cell lung cancer. Oncogene.

[16] Zhang W, Nandakumar N, Shi Y, Manzano M, Smith A, Graham G, Gupta S, Vietsch EE, Laughlin SZ, Wadhwa M, Chetram M, Joshi M, Wang F, Kallakury B, Toretsky J, Wellstein A (2014). Downstream of mutant KRAS, the transcription regulator YAP is essential for neoplastic progression to pancreatic ductal adenocarcinoma. Science signaling.

[17] de Cristofaro T, Di Palma T, Ferraro A, Corrado A, Lucci V, Franco R, Fusco A, Zannini M (2011). TAZ/WWTR1 is overexpressed in papillary thyroid carcinoma. European journal of cancer (Oxford, England : 1990).

[18] Errani C, Zhang L, Sung YS, Hajdu M, Singer S, Maki RG, Healey JH, Antonescu CR (2011). A novel WWTR1-CAMTA1 gene fusion is a consistent abnormality in epithelioid hemangioendothelioma of different anatomic sites. Genes, chromosomes & cancer.

[19] Tanas MR, Sboner A, Oliveira AM, Erickson-Johnson MR, Hespelt J, Hanwright PJ, Flanagan J, Luo Y, Fenwick K, Natrajan R, Mitsopoulos C, Zvelebil M, Hoch BL, Weiss SW, Debiec-Rychter M, Sciot R (2011). Identification of a disease-defining gene fusion in epithelioid hemangioendothelioma. Science translational medicine.

[20] Antonescu CR, Le Loarer F, Mosquera JM, Sboner A, Zhang L, Chen CL, Chen HW, Pathan N, Krausz T, Dickson BC, Weinreb I, Rubin MA, Hameed M, Fletcher CD (2013). Novel YAP1-TFE3 fusion defines a distinct subset of epithelioid hemangioendothelioma. Genes, chromosomes & cancer.

[21] Tanas MR, Ma S, Jadaan FO, Ng CK, Weigelt B, Reis-Filho JS, Rubin BP (2016). Mechanism of action of a WWTR1(TAZ)-CAMTA1 fusion oncoprotein. Oncogene.

[22] St John MA, Tao W, Fei X, Fukumoto R, Carcangiu ML, Brownstein DG, Parlow AF, McGrath J, Xu T (1999). Mice deficient of Lats1 develop soft-tissue sarcomas, ovarian tumours and pituitary dysfunction. Nature genetics.

[23] Hong JH, Hwang ES, McManus MT, Amsterdam A, Tian Y, Kalmukova R, Mueller E, Benjamin T, Spiegelman BM, Sharp PA, Hopkins N, Yaffe MB (2005). TAZ, a transcriptional modulator of mesenchymal stem cell differentiation. Science (New York, NY).

[24] Tremblay AM, Missiaglia E, Galli GG, Hettmer S, Urcia R, Carrara M, Judson RN, Thway K, Nadal G, Selfe JL, Murray G, Calogero RA, De Bari C, Zammit PS, Delorenzi M, Wagers AJ (2014). The Hippo transducer YAP1 transforms activated satellite cells and is a potent effector of embryonal rhabdomyosarcoma formation. Cancer cell.

[25] Crose LE, Galindo KA, Kephart JG, Chen C, Fitamant J, Bardeesy N, Bentley RC, Galindo RL, Chi JT, Linardic CM (2014). Alveolar rhabdomyosarcoma-associated PAX3-FOXO1 promotes tumorigenesis via Hippo pathway suppression. The Journal of clinical investigation.

[26] Eisinger-Mathason TS, Mucaj V, Biju KM, Nakazawa MS, Gohil M, Cash TP, Yoon SS, Skuli N, Park KM, Gerecht S, Simon MC Deregulation of the Hippo pathway in soft-tissue sarcoma promotes FOXM1 expression and tumorigenesis.

[27] Fletcher JAB C.D.M., Hogendoorn P.C.W., Mertens F. (2013). Pathology and Genetics of Tumours of Soft Tissue and Bone.

[28] Kanai F, Marignani PA, Sarbassova D, Yagi R, Hall RA, Donowitz M, Hisaminato A, Fujiwara T, Ito Y, Cantley LC, Yaffe MB (2000). TAZ: a novel transcriptional co-activator regulated by interactions with 14-3-3 and PDZ domain proteins. The EMBO journal.

[29] Cordenonsi M, Zanconato F, Azzolin L, Forcato M, Rosato A, Frasson C, Inui M, Montagner M, Parenti AR, Poletti A, Daidone MG, Dupont S, Basso G, Bicciato S, Piccolo S (2011). The Hippo transducer TAZ confers cancer stem cell-related traits on breast cancer cells. Cell.

[30] Coindre JM, Fletcher JAB C.D.M., Hogendoorn P.C.W., Mertens F. (2013). Grading and staging of sarcomas. Pathology and Genetics of Tumours of Soft Tissue and Bone.

[31] Dei Tos FP A.P., Fletcher JAB C.D.M., Hogendoorn P.C.W., Mertens F. (2013). Atypical lipomatous tumor. Pathology and Genetics of Tumours of Soft Tissue and Bone.

[32] Dei Tos AM-E A.P., Pedeutour F., Rossi S., Fletcher JAB C.D.M., Hogendoorn P.C.W., Mertens F. (2013). Dedifferentiated liposarcoma. Pathology and Genetics of Tumours of Soft Tissue and Bone.

[33] Hogendoorn JVMGB P.C.W., Nielsen G.P., Fletcher JAB C.D.M., Hogendoorn P.C.W., Mertens F. (2013). Chondrosarcoma (grades I-III), including primary and secondary variants and periosteal chondrosarcoma. Pathology and Genetics of Tumours of Soft Tissue and Bone.

[34] Lei QY, Zhang H, Zhao B, Zha ZY, Bai F, Pei XH, Zhao S, Xiong Y, Guan KL (2008). TAZ promotes cell proliferation and epithelial-mesenchymal transition and is inhibited by the hippo pathway. Molecular and cellular biology.

[35] Zhao B, Li L, Tumaneng K, Wang CY, Guan KL (2010). A coordinated phosphorylation by Lats and CK1 regulates YAP stability through SCF(beta-TRCP). Genes & development.

[36] Liu CY, Zha ZY, Zhou X, Zhang H, Huang W, Zhao D, Li T, Chan SW, Lim CJ, Hong W, Zhao S, Xiong Y, Lei QY, Guan KL (2010). The hippo tumor pathway promotes TAZ degradation by phosphorylating a phosphodegron and recruiting the SCF{beta}-TrCP E3 ligase. The Journal of biological chemistry.

[37] Yagi R, Chen LF, Shigesada K, Murakami Y, Ito Y (1999). A WW domain-containing yes-associated protein (YAP) is a novel transcriptional co-activator. The EMBO journal.

[38] Chan SW, Lim CJ, Loo LS, Chong YF, Huang C, Hong W (2009). TEADs mediate nuclear retention of TAZ to promote oncogenic transformation. The Journal of biological chemistry.

[39] Zhang H, Liu CY, Zha ZY, Zhao B, Yao J, Zhao S, Xiong Y, Lei QY, Guan KL (2009). TEAD transcription factors mediate the function of TAZ in cell growth and epithelial-mesenchymal transition. The Journal of biological chemistry.

[40] Zhao B, Ye X, Yu J, Li L, Li W, Li S, Yu J, Lin JD, Wang CY, Chinnaiyan AM, Lai ZC, Guan KL (2008). TEAD mediates YAP-dependent gene induction and growth control. Genes & development.

[41] Liu-Chittenden Y, Huang B, Shim JS, Chen Q, Lee SJ, Anders RA, Liu JO, Pan D (2012). Genetic and pharmacological disruption of the TEAD-YAP complex suppresses the oncogenic activity of YAP. Genes & development.

[42] Hau JC, Erdmann D, Mesrouze Y, Furet P, Fontana P, Zimmermann C, Schmelzle T, Hofmann F, Chene P (2013). The TEAD4-YAP/TAZ protein-protein interaction: expected similarities and unexpected differences. Chembiochem.

[43] Tan G, Cao X, Dai Q, Zhang B, Huang J, Xiong S, Zhang Y, Chen W, Yang J, Li H (2015). A novel role for microRNA-129-5p in inhibiting ovarian cancer cell proliferation and survival via direct suppression of transcriptional co-activators YAP and TAZ. Oncotarget.

[44] Feng X, Chen Q, Gutkind JS (2014). Oncotargeting G proteins: The Hippo in the room. Oncotarget.

[45] Feng X, Degese MS, Iglesias-Bartolome R, Vaque JP, Molinolo AA, Rodrigues M, Zaidi MR, Ksander BR, Merlino G, Sodhi A, Chen Q, Gutkind JS (2014). Hippo-independent activation of YAP by the GNAQ uveal melanoma oncogene through a trio-regulated rho GTPase signaling circuitry. Cancer cell.

[46] Yu FX, Luo J, Mo JS, Liu G, Kim YC, Meng Z, Zhao L, Peyman G, Ouyang H, Jiang W, Zhao J, Chen X, Zhang L, Wang CY, Bastian BC, Zhang K (2014). Mutant Gq/11 promote uveal melanoma tumorigenesis by activating YAP. Cancer cell.

[47] Taccioli C, Sorrentino G, Zannini A, Caroli J, Beneventano D, Anderlucci L, Lolli M, Bicciato S, Del Sal G (2015). MDP, a database linking drug response data to genomic information, identifies dasatinib and statins as a combinatorial strategy to inhibit YAP/TAZ in cancer cells. Oncotarget.

