# Supplementary material for: TAZ and YAP are frequently activated oncoproteins in sarcomas
Source: Oncotarget. 2016 Apr 25;7(21):30094–108. doi: 10.18632/oncotarget.8979 (PMC5058666; doi:10.18632/oncotarget.8979)
Supplement: Supplementary file 1 [file oncotarget-07-30094-s001.pdf]

TAZ and YAP are frequently activated oncoproteins in sarcomas

Supplementary Material

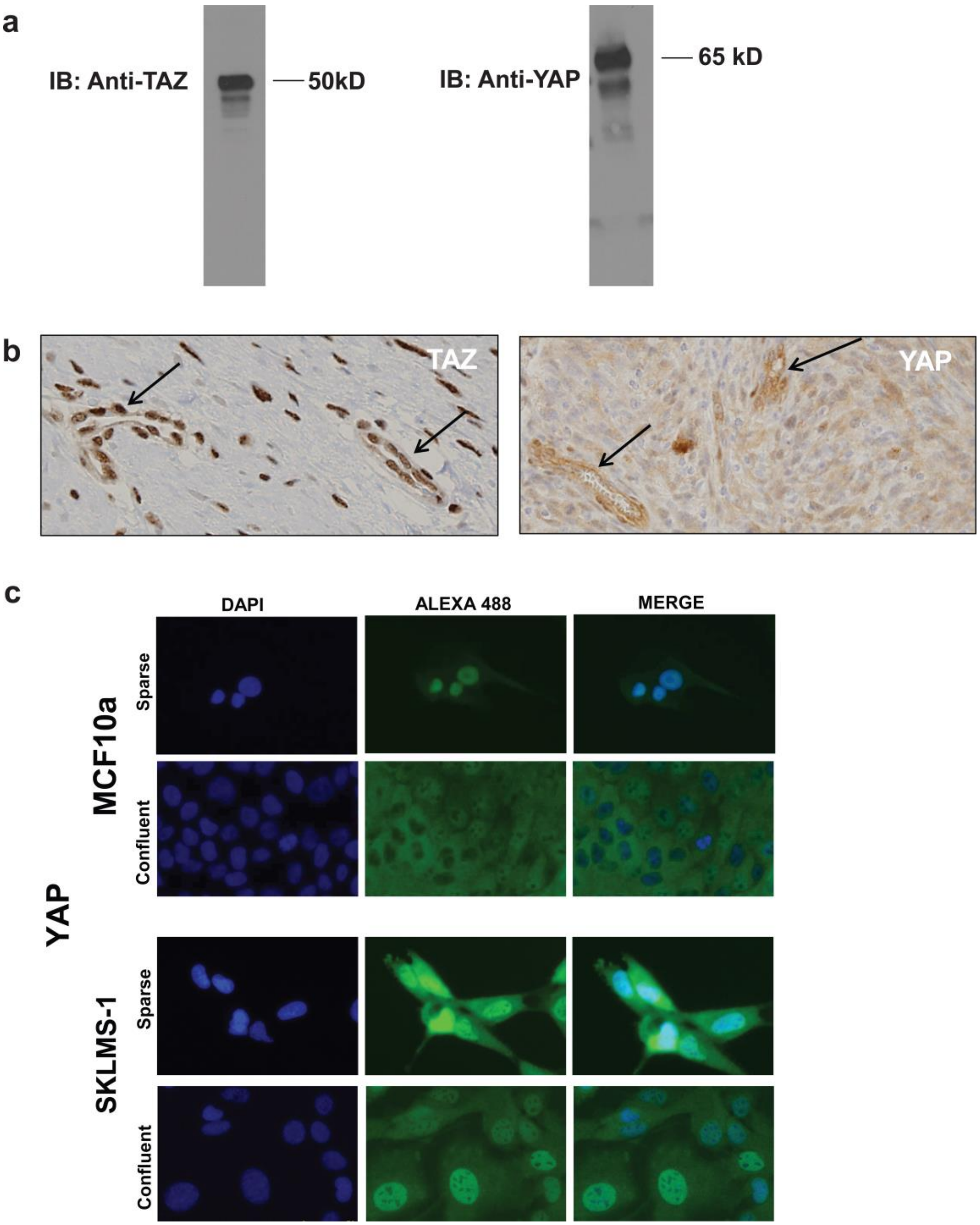

Fig. S1. **a)** TAZ antibody utilized for immunohistochemistry recognizes single band in NIH-3T3 cells transfected with pcDNA3-TAZ. YAP antibody utilized for immunohistochemistry recognizes a single band in NIH-3T3 cells transfected with pcDNA3-YAP (lower molecular weight bands represent degradation products) **b**) Intensity of staining for TAZ and YAP in the tissue microarray was determined by comparison with TAZ and YAP expression in endothelial cells (internal control in the TMAs). Endothelial cells are known to have high levels of expression of both TAZ and YAP and defined strong intensity of staining. **c**) YAP is located within the nucleus of MCF10a cells during sparse conditions, but shifts in its localization to the cytoplasm during confluent conditions due to activation of the Hippo pathway. In contrast, YAP remains localized within the nucleus of SK-LMS-1 cells during both sparse and confluent conditions since it is no longer being inhibited by the Hippo pathway.

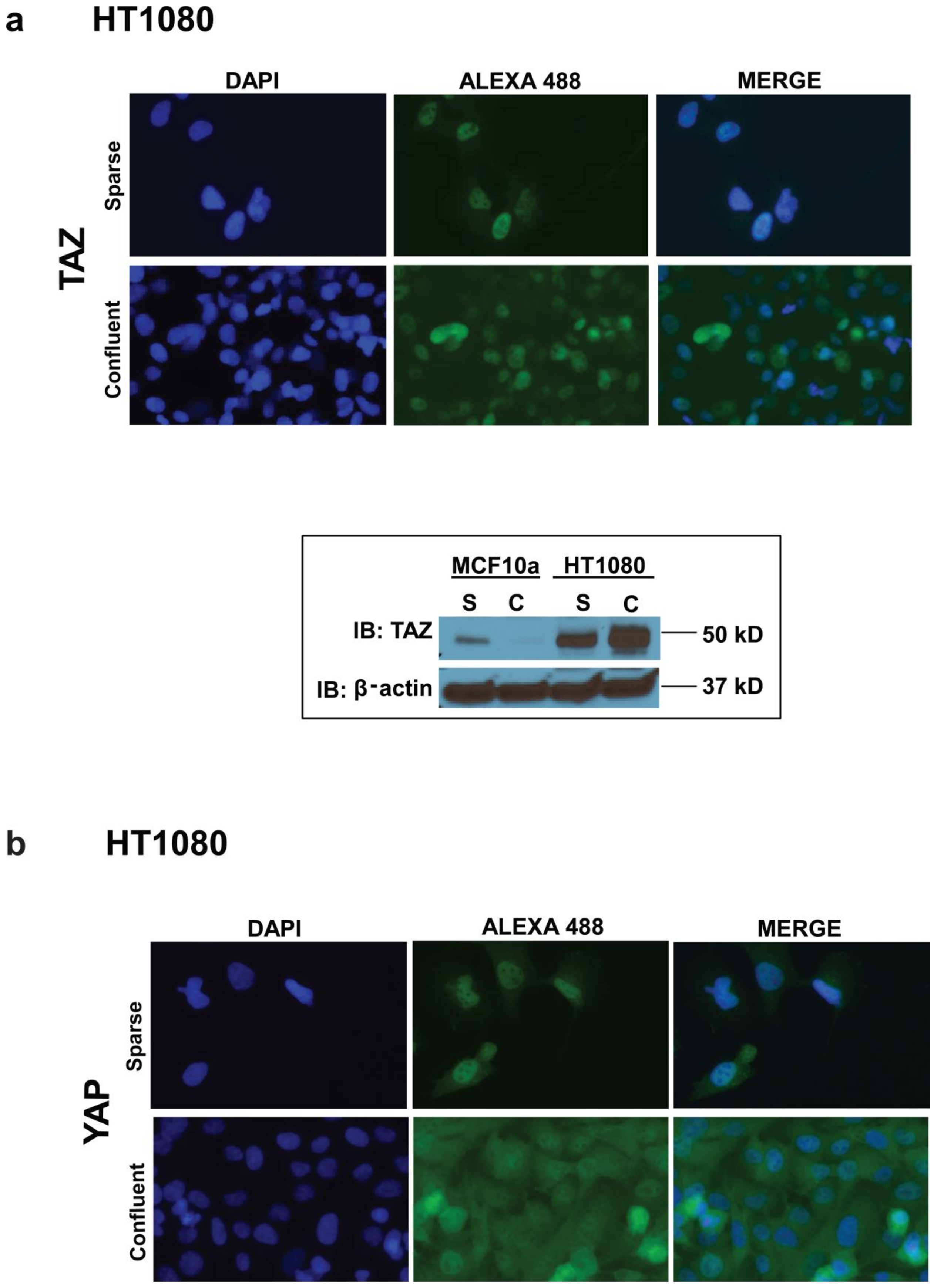
a
HT1080
DAPI
ALEXA 488
MERGE
TAZ
Sparse
Confluent
MCF10a
HT1080
S
C
S
C
IB: TAZ
IB: β-actin
50 kD
37 kD
b
HT1080
DAPI
ALEXA 488
MERGE
YAP
Sparse
Confluent

Fig. S2. TAZ and YAP localization in HT-1080 cells during sparse and confluent conditions. **a**) TAZ is localized within the nucleus of HT-1080 cells during both sparse and confluent conditions. As demonstrated before, western blot demonstrates that levels of TAZ in MCF10a cells decrease when cells are grown to confluence. However levels of TAZ in HT-1080 cells are relatively constant during confluence because TAZ remains localized within the nucleus since it is constitutively activated. S=sparse, C=confluent for Western blot. **b**) YAP is localized within the nucleus in HT-1080 cells during both sparse and confluent conditions, consistent with its constitutive activation and lack of inhibition by the Hippo pathway.

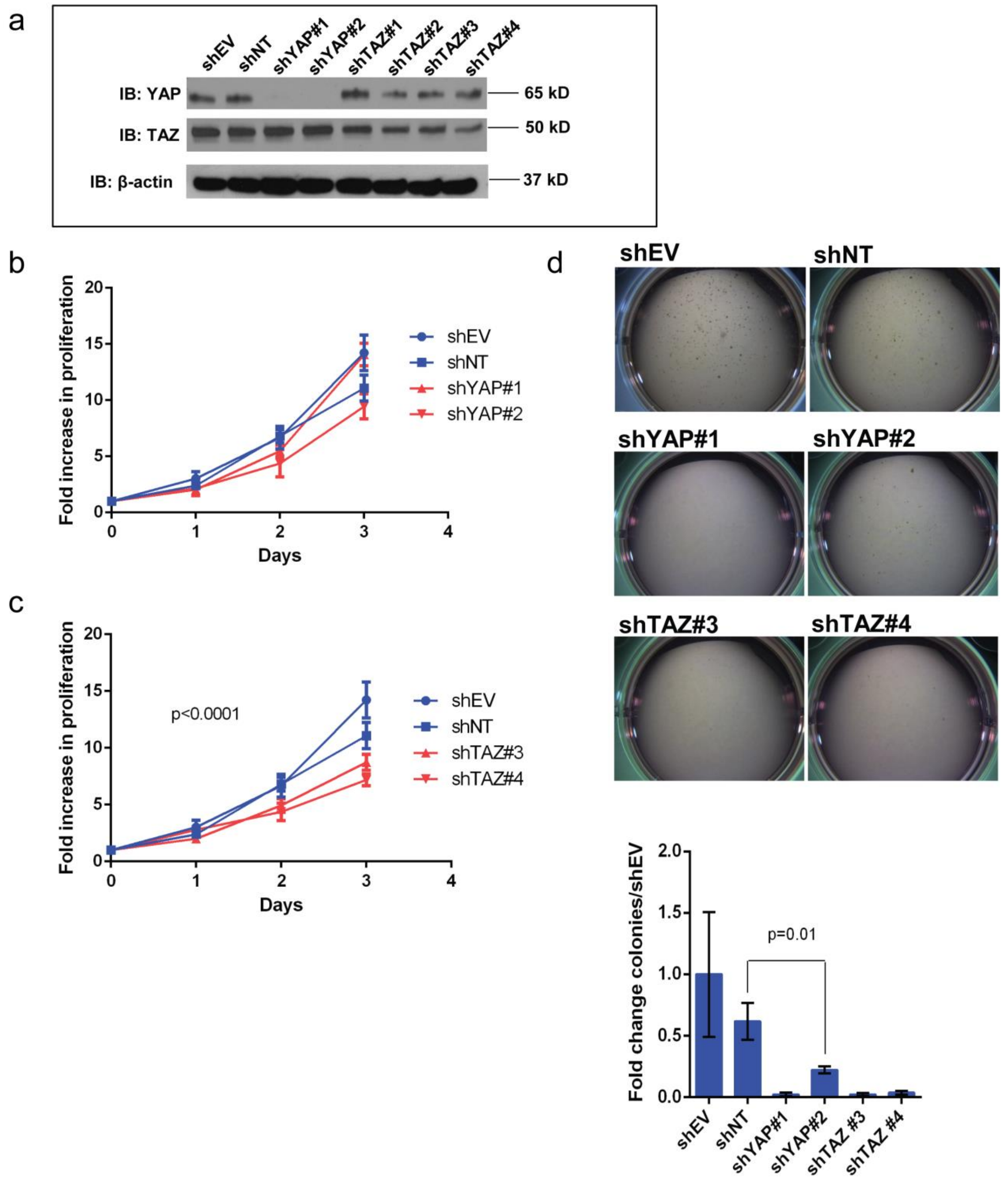

Fig. S3. **a**) YAP and TAZ are effectively knocked-down in HT1080 cells with multiple shRNA constructs (shTAZ #3 and #4 produced the best knockdown and were used for subsequent experiments) **b**) Knock-down of YAP did not significantly change proliferation. **c**) Knock-down

of TAZ significantly reduced proliferation ($p<0.0001$). d) Knock-down of YAP or TAZ abrogate colony formation in soft agar ($p=0.01$).
